# Unmanned Aerial Vehicle Based Wireless Sensor Network for Marine-Coastal Environment Monitoring

**DOI:** 10.3390/s17030460

**Published:** 2017-02-24

**Authors:** Carlos A. Trasviña-Moreno, Rubén Blasco, Álvaro Marco, Roberto Casas, Armando Trasviña-Castro

**Affiliations:** 1 Instituto de Investigación en Ingeniería de Aragón, Universidad de Zaragoza, Zaragoza 50018, Spain; rblasco@unizar.es (R.B.); amarco@unizar.es (Á.M.); rcasas@unizar.es (R.C.); 2 Centro de Investigación Científica de Educación Superior de Ensenada Unidad de La Paz, La Paz 23050, Mexico; trasvi@cicese.mx

**Keywords:** remote sensing, WSN, UAV, LPWAN, LoRa, marine monitoring, low power electronics

## Abstract

Marine environments are delicate ecosystems which directly influence local climates, flora, fauna, and human activities. Their monitorization plays a key role in their preservation, which is most commonly done through the use of environmental sensing buoy networks. These devices transmit data by means of satellite communications or close-range base stations, which present several limitations and elevated infrastructure costs. Unmanned Aerial Vehicles (UAV) are another alternative for remote environmental monitoring which provide new types of data and ease of use. These aircraft are mainly used in video capture related applications, in its various light spectrums, and do not provide the same data as sensing buoys, nor can they be used for such extended periods of time. The aim of this research is to provide a flexible, easy to deploy and cost-effective Wireless Sensor Network (WSN) for monitoring marine environments. This proposal uses a UAV as a mobile data collector, low-power long-range communications and sensing buoys as part of a single WSN. A complete description of the design, development, and implementation of the various parts of this system is presented, as well as its validation in a real-world scenario.

## 1. Introduction

Ecological monitoring is an area of great interest for remote sensing, mainly due to the increased concern for the preservation of the environment, as well as the impact of climate change and human activity. Diverse implementations have been widely used in fields such as forest monitoring [1,2], wildlife preservation [3,4] and agriculture monitoring [5,6].

Another field in which remote sensing has played a key role in recent years is the marine environment. It is probably one of the most important areas in ecology, as changes in this environment directly impact the biodiversity and atmospheric conditions, as well as any fishing-related activities [7,8,9]. Moreover, it is also one of the harshest settings to monitor for any type of deployment, as it requires waterproof robust technology to endure the high levels of humidity and salinity, wave collisions, and extreme weather conditions [10]. The way in which marine environments have been monitored has changed little in the past few decades, with the majority of implementations being based on satellite imagery, underwater devices with various sensors, and buoy developments. The latter are by far the most commonly used, being mostly applied to ambient variable measurement, either as stationary or drifting devices, each with its own specific purpose. These devices can measure several variables simultaneously and monitor areas for long periods of time. Currently, there are at least 1354 stations and 1421 drifter buoys deployed globally, according to data from the National Data Buoy Center [11] and The Global Drifter Program [12] correspondingly. Yet one of the main drawbacks of using buoys is the process for data extraction. The most commons methods are long-range communications, close-range wireless transmissions, or dry-land data collection. Long-range transmissions require either a previously established infrastructure, such as a ground base station, or satellite connectivity. When using a ground station, the buoys must be fixed to a given position, or several stations must be established for drifting buoys. Additionally, these types of wireless communications tend to have a range limited to less than 100 m per ground station, thus the high usage of satellite communications. In the case of short-range communications and dry-land data collection, an aquatic vessel is required to reach the buoys and either extract the data or the buoy itself. All the previously mentioned methods are hardly sustainable due to expenses associated with monthly fees, maintenance, or person-hours.

Currently, one of the most novel approaches for monitoring marine environments is the use of Unmanned Aerial Vehicles (UAV). These devices can cover areas several km wide, given their long-range communications, enabling the possibility of monitoring hard to reach areas with relative ease, as well as acquiring similar data than that of satellite imagery. In this last area, its contributions highly benefit from the possibility of extracting data at a higher resolution than satellites, but in contrast they may only cover smaller areas of analysis [13]. Additionally, as they are airborne devices, UAVs do not face the same challenges as marine monitoring deployments. Implementations using these types of devices have been used for wildlife [14,15,16,17], environmental [18,19], and hazardous marine monitoring [20]. Usually the data type acquired by a UAV in a marine implementation are those that can be collected using infrared, multispectral, or conventional cameras, yet environmental data are, for the most part, unattainable. Moreover, given their short life span of tens of minutes and the need for user interactions, monitoring for extended periods of time is difficult to achieve, as it would require several landings and battery replacements.

To overcome the limitations of UAV and buoy based implementations, the use of a hybrid Wireless Sensor Network (WSN), which uses both technologies, is proposed in this article. This type of UAV assisted WSN system architecture has become increasingly used in the past few years, given recent advances in both areas. In the agricultural sector, these type of implementations have been used to monitor crops remotely, by using a UAV as a data collector [21,22] and, in some cases, as a trigger for countermeasures against pests [22]. Similarly, the use of UAVs has been proposed as a means of managing disaster situations, in conjunction with both WSN [23,24] and cellular networks [24,25], which presents several improvements over current implementations. In other areas, such as the entertainment sector, UAV assisted WSN have been proposed to provide enhanced user experiences in various types of events, such as music concerts or sports [26].

In aquatic environments, similar approaches as the one proposed in this article can be found in the literature, such as the work of Zolich et al. [27] and Barbatei et al. [28]. Zolich et al. propose the use of a UAV to collect data from stationary low-cost and low-power consuming buoys, which provide data regarding various underwater sensors. To transmit data to the UAV, they used an 868 MHz radio module which implements the Tiny Mesh protocol, with a power consumption of 24 mA in reception and 560 mA in transmission, both at 3.3 V. In their experimentation, they were able to transmit with a maximum data rate of 4399.97 bps and a data link range of 485 m, whilst the UAV was hovering at approximately 9 m above sea surface. Barbatei et al. use a similar system architecture with a UAV as a data mule and custom designed modular stationary buoy devices, which house a temperature sensor, an Inertial Measurement Unit (IMU), GPS module, micro SD storage, and a wireless transmitter, all managed by a 16 bit microcontroller. The wireless transmitter used in their implementation is a Radiocrafts RF1280 module, which transmits at 868 MHz frequency with a FSK modulation with an estimated data rate of 4.8 kbps. According to their study, this provides an approximate range of communications of 264 m with the UAV, with a low current consumption of 21 mA in reception and 28 mA in transmission, and a 3.3 V voltage supply.

The system proposed in this article aims to use a swarm of drifting buoys, contrary to static ones presented in the previously mentioned work, as primary environmental sensors, covering wide areas of seashore, and a UAV as a data sink and dynamic network router. The use of drifting buoys makes the use of short-range communications with the UAV completely unviable, thus, to be able to locate and extract the data with a UAV, it is desirable to have long-range communications to simplify their interaction. The selection of a proper wireless communication protocol to fully integrate these devices is paramount. There are several possibilities for these type of transmissions, yet the recent development of the Low Power Wide Area Network (LPWAN) protocols present new opportunities with the benefits of a reduced energy consumption and moderate data rate [29,30]. One of the most promising technologies in LPWANs is the LoRa spread spectrum modulation, which has been proven to be able to communicate data across several kilometers with a low power consumption [30,31,32,33]. The unique modulation of this technology not only offers the possibility of transmitting below the noise floor, but has also a high resilience to external interference [34]. As a slight drawback, similar to most LPWANs, the LoRa technology presents a limited transmission bitrate, which is sufficient for most sensing applications although not ideal for high data streaming. Having stated the capabilities of the LoRa modulation, as well as the requirements of the application, it can be seen that it seems to properly suit the necessities of this implementation, avoiding the use of satellite or GSM communications, as well as additional cumbersome infrastructure.

In the following sections of this paper a full description of the proposed system and its functionalities are encompassed. Afterwards, from both a hardware and firmware perspective, the architecture of the system is analyzed, as well as the custom network layer protocol designed for this implementation. Finally, the description of a series of trials, their methodology, a field test for the validation of the system, a discussion of the results, and the final conclusions are explained.

## 2. SIMMA Functional Overview

The implementation of this multi-device solution was sought out to cover the necessities of the SIMMA research project in Mexico. The aim of this project is to monitor different variables in the Mexican shoreline, using low-power and low-cost sensing buoys which can be scattered across a wide area. The buoys, as stated before, are to be used mainly as drifting devices, although a scenario where they are used as stationary buoys, by anchoring them to the seafloor, has also been contemplated within this project. To be able to access their data, a UAV is to be used as a mobile data harvester and network manager. By design, UAVs can be controlled across great lengths using different wireless communications, using mostly different types of UHF transmissions for video and flight data feeds, as well as flight controls. To be able to act as a network router, it was important to select a wireless communication technology which would not affect the operation of the UAV, as any interference could have catastrophic consequences. One of the key characteristics of LoRa is its unique modulation, which allows its coexistence and operation with other RF technologies presumably without interference [34], thus the use of this type of transmission seems adequate for its interaction with the UAV and its multiple radio modules.

**Figure 1:**
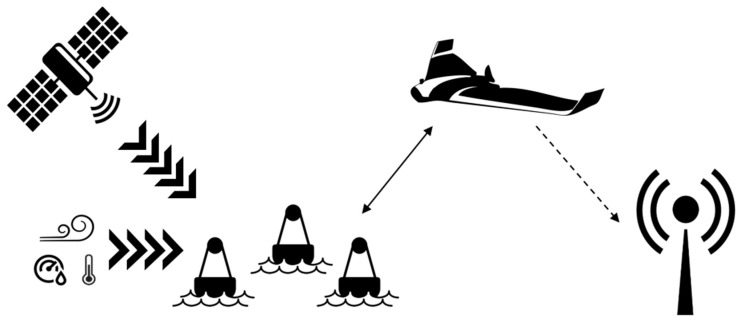
System interaction amongst the different devices of the network. The straight-line arrow indicates the main data transmission flow, the dashed arrow represents the communications with the optional ground station and the others indicate the environmental input to the buoys.

Under the SIMMA project, there are two main functional scenarios: data extraction and search and rescue. In both cases, the buoys will be periodically collecting data regarding water temperature, wind speed and its GPS coordinates, as well as air temperature, pressure, and humidity. In the data extraction scenario, after the buoys have been set on the shore line and ambient variables have been collected, the UAV will fly in the last known direction of the buoys, periodically searching for them. Once the buoys have been found, the UAV will start transmitting data acquisition messages to collect the data stored in the buoys. After the completion of this task, or if it starts running low on battery, the UAV will return to home. Given that the buoy recruiting and data collection process is fully automated, to be able to monitor the network activity of the SIMMA system, a secondary optional ground station can be set on the beach. This ground station will not interact with the UAV, it is only meant to be used as a network analyzer or sniffer (Figure 1).

In the search and rescue scenario, the UAV will be used to locate missing buoys that may have drifted away from the rest of the swarm. Given that the buoys may be either stranded or in a distant location, the UAV will be flown in the suspected direction of the buoys and will try to collect the position of any buoy in its path before its return to home. This functionality simplifies the recovery of the buoys, which, in many cases, are lost to strong currents or vandalism [10,35,36].

## 3. SIMMA System Architecture

**Figure 2:**
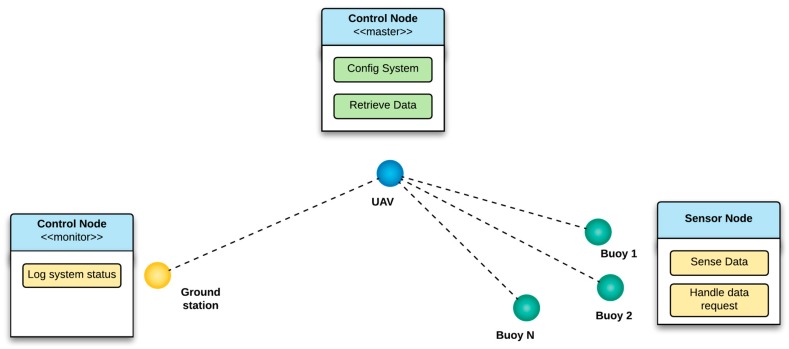
Functionality of the hardware developed for the SIMMA project.

As stated previously, for this proposal a multi-device solution was implemented to try and cover the different necessities of the SIMMA project. Two distinct hardware were developed with LoRa wireless communications: the control node and the sensor node. The control node functions either as a network master, mounted on the UAV, or as a network monitor in a ground station; the sensor node hardware can only be placed inside the buoy (Figure 2).

For both hardware, a firmware was developed with a custom network layer protocol which will be referred to as LoRaNET. Additionally, to be able to interact with the SIMMA system, a Graphical User Interface (GUI) was also developed to simplify the process of configuration and mode switching. When using a base station as monitor, the GUI may also be used to display the system’s activity. The design and implementation of the previously mentioned hardware, firmware, network layer protocol, and GUI were developed by the authors at the Universidad de Zaragoza, Spain.

Given the complexity of the system, and to better explain the full functionality of this proposal, this section has been divided in various subsections covering the electronic hardware, software, firmware, and the GUI. Moreover, the description of the UAV and buoys used in the implementation is also covered.

### 3.1. LoRaNET

The implementation of a design specific network layer protocol was sought out given that the current LoRa protocol, LoRaWAN [37], does not include the functionality for the transceiver in the 433 MHz frequency band, which is the operating frequency that is to be used for the SIMMA project. Moreover, there is less flexibility while configuring the LoRa settings which may increase the transmission range, as well as a high packet overhead which incurs directly in the power consumption.

LoRaNET is a proprietary network layer implementation, designed for this specific proposal, which uses the LoRa modulation, oriented for ultra-low power WSN, where the synchronization needs and bandwidth are not elevated. LoRaNET is able to implement a star network topology and defines two simple types of nodes:

- Master node: Creates and manages the network, assigning network addresses to the other nodes and centralizing the message traffic;
- Slave node: Any other node that is part of the network.

Every time a LoRa network is created, a series of basic parameters are established for its functionality. Within these parameters are the network ID, the maximum wait time for an answer within the network, the maximum wait time for a node to leave the network after the last message, and the number of attempts a message can be re-sent if there is no acknowledgment of message reception. These parameters must be adjusted accordingly with the LoRa RF settings, which alters the transmission range, as the time on air of a message may vary greatly.

Once the network has been created, the master node can recruit any slave node within its reach as long as it is not part of any active network. If a message can be answered by several slave nodes simultaneously, as when recruiting, scanning the network or with any type of broadcast message, the answer is sent in pseudo-random intervals, reducing the possibility of RF packet collisions.

When a slave node has been recruited, the master assigns him a 2 byte unique ID in the network, which is associated with the device’s MAC address. If for any reason a slave gets disconnected, upon reconnection it will be assigned the same ID. The master node will always be assigned the 0x0000 address, and the 0xFFFF address is reserved as a broadcast identifier.

In the Table 1, a summary of the main commands implemented in the current version of LoRaNET is shown.

### 3.2. Sensor Node

**Figure 3:**
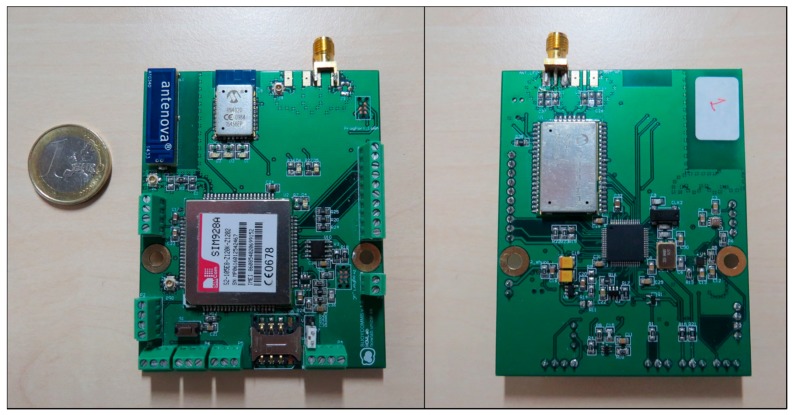
Design of the PCB for the environmental sensing buoys.

For this part of the implementation, a custom designed PCB was employed. The main processing unit is a 16 bit PIC24FJ128GC006 microcontroller [38], which manages all the peripherals and communications. Given its architecture, it offers several low power configurations that can be used to increase the life span of the device. For the wireless communications the RN2483 transceiver was selected [39], as it offers the possibility of transmitting data in a wide range of frequencies with the LoRa modulation. Additionally, the board was suited with an 8 Mbit flash memory and a SIM928A GPS and GSM integrated circuit [40] (Figure 3).

It is worth noting that these PCBs, as shown in Figure 3, have additional devices integrated in the board, such as a Bluetooth modules, accelerometers and SIM card ports for the GSM module. These ICs and modules, although not currently used, are intended for future use and other functional scenarios, providing additional functionality to the boards.

**Figure 4:**
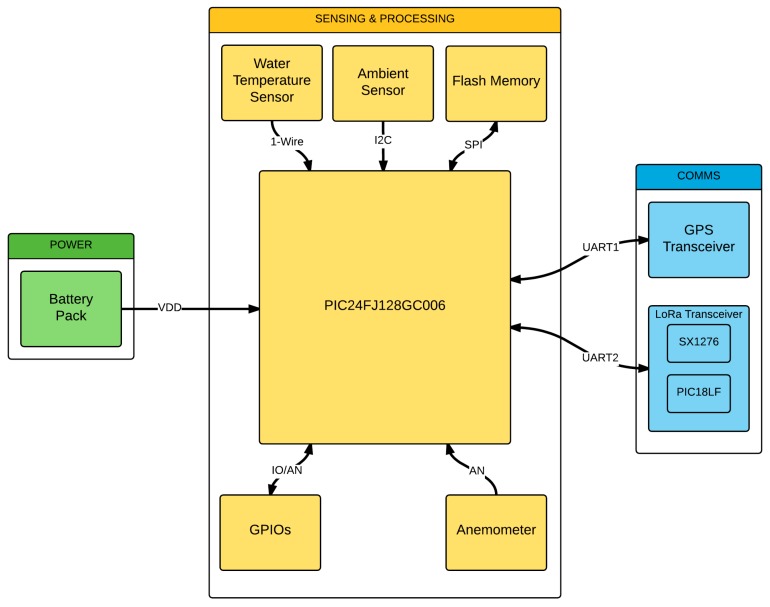
Block diagram of a sensor node’s peripheral connections.

Given that these PCBs are intended to be used as ambient sensor nodes, an external BME280 sensor for measuring air temperature, humidity, and pressure [41] was included. Additionally, they also incorporate a 1773 anemometer [42] and an insulated DS18B20 temperature sensor for water measurements [43]. These devices are all connected to the PCBs through the different terminal blocks present in the design, and are managed by the microcontroller through I2C, One-Wire, or digital communications (Figure 4). Moreover, the sensor nodes were coupled with a 433 MHz omnidirectional passive antenna with a 50 ohm impedance.

**Figure 5:**
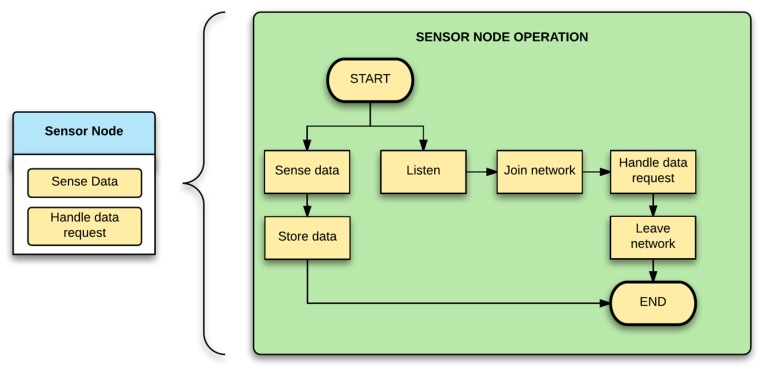
Sensor node’s task diagram.

The sensor node functionality can be summarized in two high level tasks: sensing data and handling requests. The first of the tasks is the simpler of the two and will be carried out periodically, depending on how the node is configured. The handling request task manages all the LoRa incoming messages, whether it is a data transmission or configuration activity (Figure 5).

Within the configuration activities, the sensor node’s LoRa RF settings and the device´s operating configuration can be set by the master node through the LoRa communications. The first of these are used to modify the transceiver’s internal registers, which alter the transmission frequency, spreading factor, coding rate, and channel bandwidth [44,45]. These settings will determine the range and data rate at which transmissions will be made, as well as the level redundancy used to add robustness to the transmission and avoid interference that could be found in the environment. On the other hand, the device operation configurations are the ones that implement the buoys’ functionality, which includes the data capture frequency and the time period in which the LoRa transceiver will be turned on. This last feature is thought for scenarios where the buoys will be left for several days at the sea, and the data collection with the UAV would be done periodically at certain hours of the day. This helps reduce the energy consumed by the sensor node and, in consequence, extends its lifespan. All of the sensor nodes can be reconfigured to the initial start-up settings, either by command input or a physical hardware reset button.

As the sensor node is thought to work for extended periods of time, low power consumption modes were directly implemented. Although these modes are always present, the longevity of the devices will be directly impacted by the data capture frequency. At higher frequency intervals, the device will capture almost complete streams of data, but will only last a short period of time and vice versa.

### 3.3. Control Node

**Figure 6:**
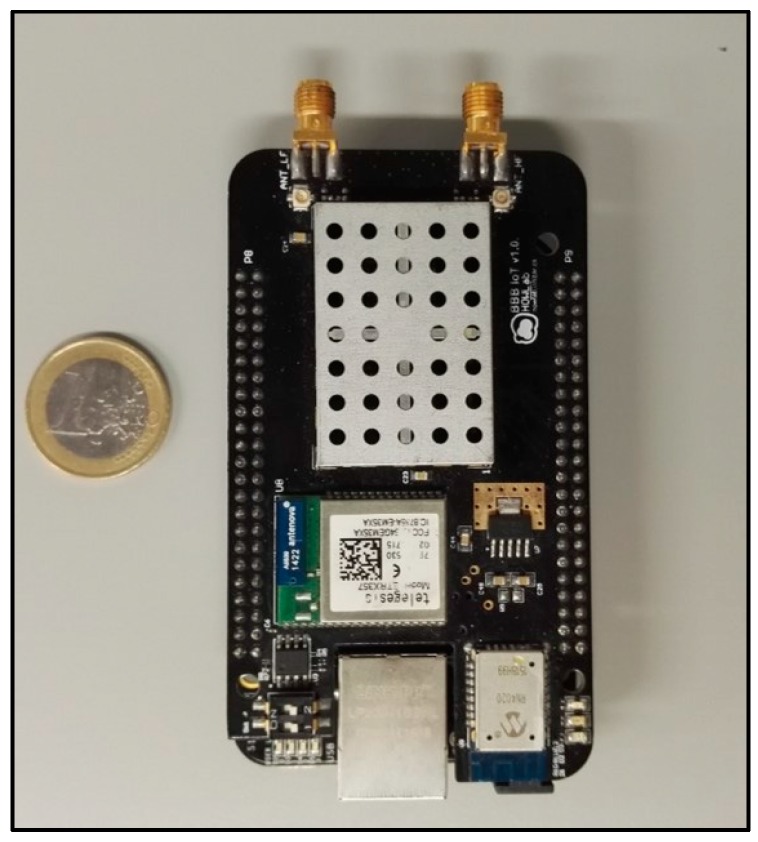
Control node: BeagleBone Black and IoT cape.

The hardware for this device was based on the BeagleBone Black (BBB) board [46], which has an AM335x 1 GHz ARM^®^ Cortex-A8 microprocessor, 512 MB of DDR3 RAM and 4 GB of 8-bit eMMC on-board flash storage. The BBB was selected, given that the control node is meant to function as a network gateway in an IoT scenario. Its communication capabilities have been augmented by integrating a custom designed cape (IoT cape), which has a built in RN4020 Bluetooth module [47], an ETRX357 Zigbee module [48], and a LoRa SX1276 transceiver [49]. With the inclusion of the IoT cape to the BBB design, an intelligent device that could manage various WSNs and link them to the IP world was created (Figure 6).

**Figure 7:**
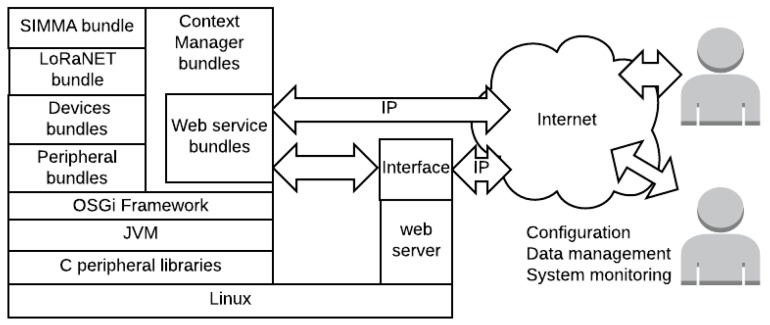
Control node software architecture.

Its programming has been implemented following a modular approach based on the Open Services Gateway initiative (OSGi) [50] (Figure 7). OSGi defines a framework where pieces of code are organized into bundles that can be managed separately (e.g., installed, updated, removed, etc.). These bundles are agents which might be dedicated to specialized tasks, such as handling a serial port or providing a command line interface. Moreover, they communicate and interact with each other by means of services which are published within the framework, which are available to each bundle.

Although several bundles are implemented in this application, to better understand the functionality of this device, the following must be described:

- Peripheral and device bundles: Enable the usage of the diverse communication transceivers present in the IoT cape, publishing them in the framework. They are composed by two layers: the peripheral and the device layer. The former gives access to the ports and communication interfaces—such as GPIOs, UARTs, SPI and I2C—so that they may be accessible from the framework. The former provides the methods to configure and manage each of the transceivers;
- LoRaNET bundle: Implements the LoRaNET network layer, providing the methods to manage or connect to an existing network;
- SIMMA bundle: Defines the application layer and the main functionality of the device;
- Context Manager (CM) bundle: Manages and stores the data originating from the sensor nodes, guaranteeing its allocation in the data base, as well as its access from external systems through a Representational State Transfer (RESTful) API or the direct data processing in the platform. This same API has been enhanced to enable control, configuration and notification of events from a remote interface (external to the OSGi framework).

Additionally, this device also functions as a UI which accesses the historical data of the sensor nodes, through the CM, to convey commands or configure the system.

**Figure 8:**
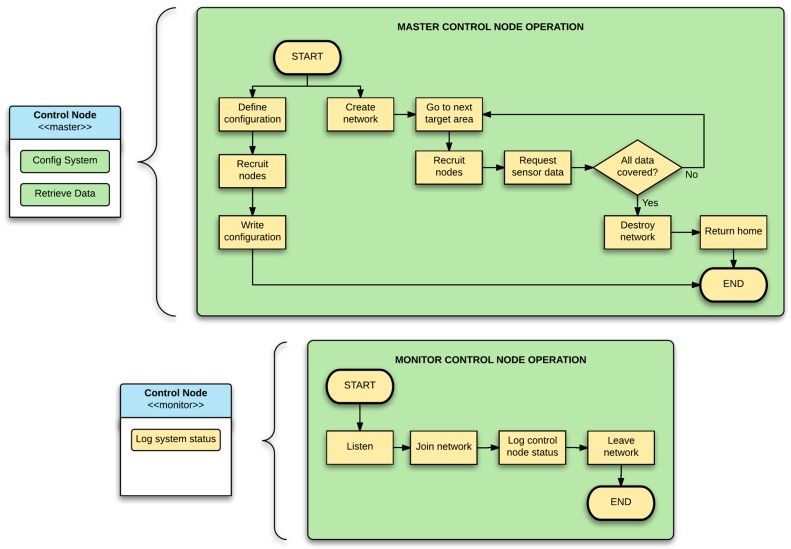
Control node’s task diagrams.

Depending on their role, the control nodes can be configured either as masters or monitors. A master node is responsible for creating and maintaining the network, assigning network addresses to the other nodes and centralizing the message traffic. The master node functionality can be summed up in two types of tasks, one for configuring the system and another for retrieving data. In the former, the master node is able to change the LoRa RF settings of the network it will create, configure the sensor nodes with these same settings and modify the sensor nodes operating configuration, mentioned in the previous section. In the latter task, the master node is able to retrieve relevant data from the sensor nodes and store it in its memory. The monitor nodes, on the contrary, may only function as information providers to the users, with the single task of reporting the activities of a master node when it is onboard the UAV without any interactions (Figure 8).

### 3.4. Buoy Design

**Figure 9:**
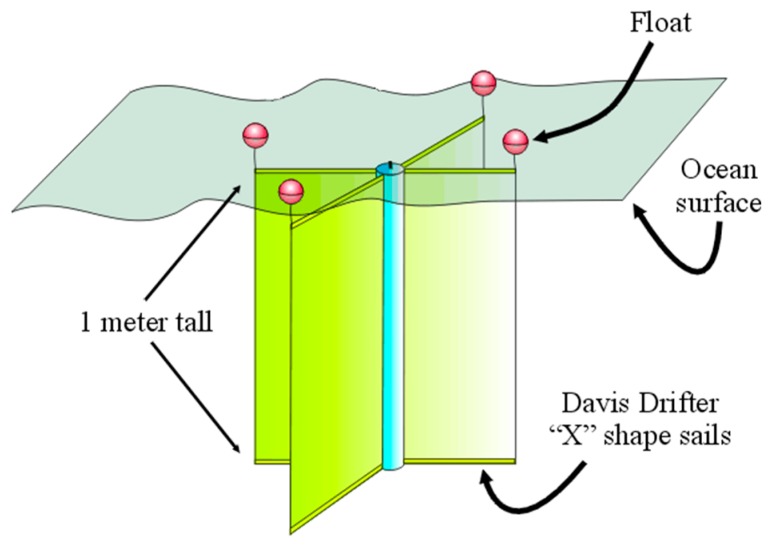
Davis drifter buoy design. Modified from http://www.ims.uaf.edu/NPRBdrifters.

The buoys are based on a design of the Davis drifter buoy, which has an “X” style sail and four floatation devices. This design is commonly used in oceanographic surface monitoring, given its ease of implementation and the ability to use the surface layer water currents to move (Figure 9).

The central structure of these particular buoys was made of hydraulic PVC, the sails from sheets of plastic canvas and the flotation devices from rigid styrofoam. These buoys were built and developed by the Centro de Investigación Científica y Educación Superior de Ensenada (CICESE) from Mexico, which is part of this project.

**Figure 10:**
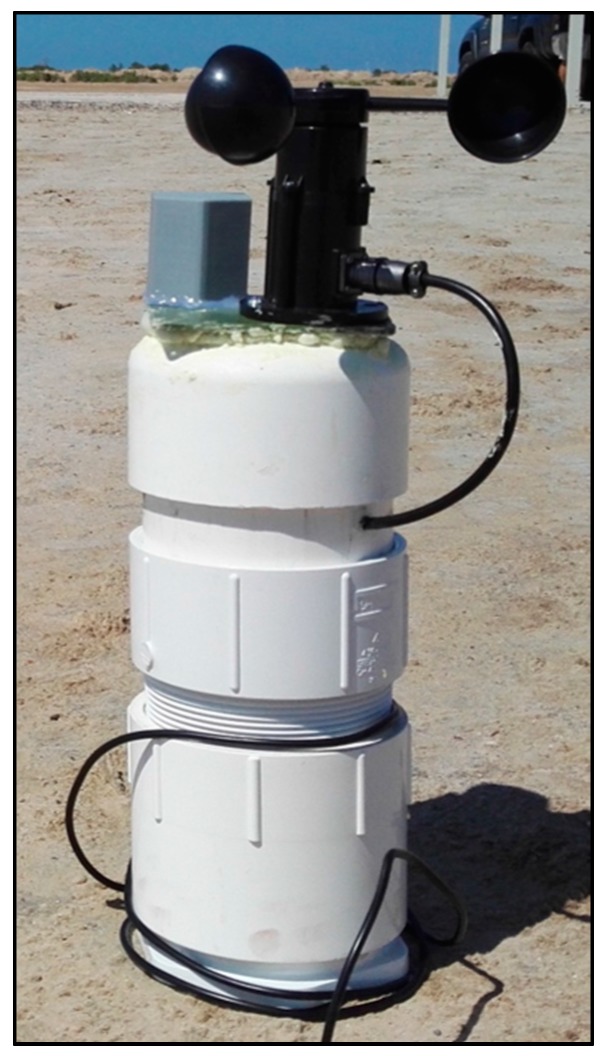
Upper part of the buoy where the electronics are housed and the external sensors are fixed.

In the upper central part of the buoy, an additional PVC tube was added to the central structure, where the slave node´s electronics were housed. Furthermore, the anemometer was fixed at the top, as well as the BME280 sensor which was contained inside of a 3D printed housing which allowed the flow of air while avoiding any water intakes to get through to the sensor. A proper air exchange is required for an accurate reading of the BME280, otherwise the sensor will measure different values than that of the actual ambient. This upper structure of the buoy will be floating above the surface of the water, allowing GPS and LoRa communications (Figure 10).

Given that this upper section of the buoy contained electronics, it was extremely important to avoid any water intakes. To try and circumvent this, the electronics were also placed in an IP67 case, and all of the connection cables for the exterior sensors that had to pass through the PVC structure were sealed with a waterproof silicon-based resin.

### 3.5. UAV Design

**Figure 11:**
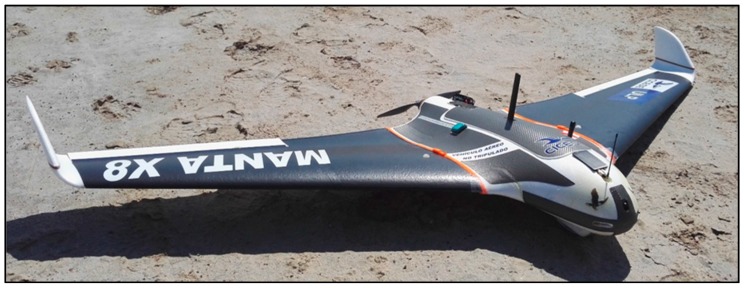
Delta Wing UAV used in the SIMMA project.

The UAV is based on a Delta Wing design, built with a foam frame with two carbon fiber rods across the wings for reinforcement and with a wingspan of 2 m. This type of design is ideal for remote areas, as it does not require a specific landing area, due to its belly pan landing system, and it is light enough (4.2 kg with battery and electronics) to be launched by hand or a small launchpad (Figure 11).

The UAV is equipped with a Panda II autopilot system kit [51], which includes a GPS receiver module, three-axis MEMS gyros, three-axis accelerometer, three-axis magnetic sensor, a barometric pressure sensor, and an airspeed meter. This system provides a high precision flight control with three different operation modes: manual, AFSS activated (active stabilization), and autopilot. The autopilot can be controlled and configured with the use of the GCS software provided by the manufacturers. This software provides a graphical interface which provides real-time data for various flight parameters of the UAV and an electronic map for waypoint navigation. To communicate the GCS with the UAV, an FY-605 wireless data radio at 462.125 MHz is utilized [52], providing an extended transmission range of up to 15 km. Moreover, the UAV has a 900 MHz First Person View (FPV) video transmitter, with its corresponding integrated camera, and a UHF Long Range System (LRS) receiver at 455 MHz. These last two wireless modules are used to control the UAV in manual mode with a radio controller.

Taking into consideration the UAV’s battery weight, servos, and electronic systems previously mentioned, the aircraft is able to carry approximately 600 g of payload. Given that the master node, its housing, and battery pack weigh roughly 360 g, the UAV is able to fly a maximum of 40 min, with the possibility of adding further peripherals. It is worth mentioning that this device was also provided by the CICESE Institute.

### 3.6. SIMMA Graphic User Interface

**Figure 12:**
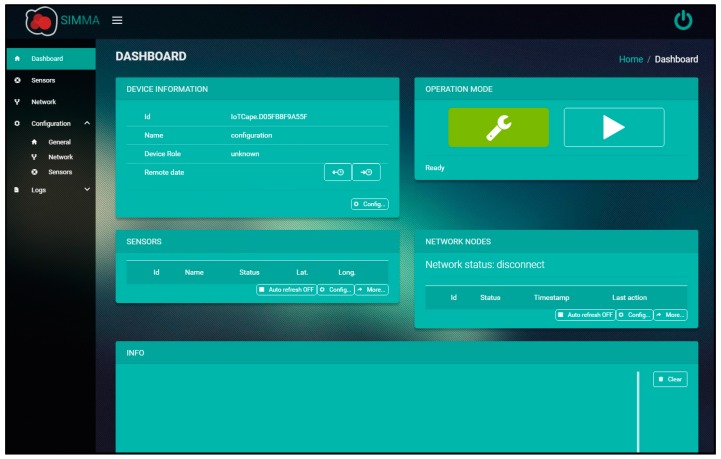
Graphical user interface screenshot.

The GUI is a single page web application built with Angular 2 [53] that was designed specifically for its use in this project as a configuration and visualization tool. The selection of a web-based interface was sought out to avoid installing custom software on the client´s equipment and, thus, reducing the compatibility issues amongst different operating systems, as well as providing a self-contained solution (Figure 12).

To access its features, the control node must be connected through a network interface and afterwards, in a web explorer, access its predefined IP address. As the BBB provides connectivity through a Virtual Ethernet Port interface over USB, it is possible to connect this device through this serial port to any computer, simplifying its usage.

Having accessed the interface, the user is able to configure a control node as a master or monitor. In master mode, the functionality of the GUI becomes far more complex, as it allows the user to not only configure the master node, but also the sensor nodes through interactions with the master. There are two modes of operation for the master node: manual and automatic. For configuration in a laboratory or a ground station, the manual mode must be selected, as it allows the user to set the LoRa RF settings of the master node and slave nodes (sensors and monitors), as well as the operation settings of the sensor nodes. Moreover, in this mode the user can manage the data from the sensor nodes, downloading or deleting them, and graphically visualize all the downloaded sensor data, as well as exporting them in several formats (xml, json, csv, or kml). The automatic mode is used when the node is to be mounted on the UAV, having previously configured the rest of slave nodes with the same RF settings.

When in monitor mode, the user is able to view the complete message log of the master node in real-time, including the localization of sensors nodes, network activities, and data extraction process. To be able to view a given master node, the monitor node must also be configured with the exact same LoRa RF configurations.

## 4. Experimentation and Results

### 4.1. Objectives

The main objective of a system field test, as with any application, is to validate its complete functionality and identify possible improvements. Yet, given the complexity and amount of device interactions in the SIMMA system, several tests were required prior to its validation as a whole. The objectives of these tests were to:

- Validate the LoRa wireless communications amongst the control nodes and sensor nodes;
- Verify the proper stability and functionality of the control nodes and sensor nodes;
- Analyze the LoRa communications behavior in a similar environment to the final field test, and its interaction with the UAV’s transceivers.

### 4.2. Methodology

As the LoRa communications play a key role in the system, it was one of the first things that needed to be tested. As stated previously, the LoRa modulation has been widely studied and its achievable range proven, yet its operation at 433 MHz has not been analyzed in detail. This analysis, and a comparison with the 868 MHz band, was done in a previous study [32], where several RF configurations were tested with a beta version of the sensor node communications block. From this experimentation, several LoRa RF configurations were selected for further technical testing.

With the control and sensor node hardware used in this article, stress and stability tests were done in a laboratory. Periodically, the sensor node would capture data from all the ambient sensors and GPS module, store the data in its memory and await LoRaNET messages. The control node was configured to automatically create a network, search and recruit sensor nodes, and download their data. This was accomplished with the master node connected directly to the GIU. These tests were done several times, after fixing the identified coding errors, until the firmware and software of the devices were stable for the next trials.

To accomplish the third objective, several experimentations were deemed necessary. The first trial was done by transmitting a sequence of data packages across a shore line with both transceivers at sea level, trying to emulate LoRa transmissions in the worst-case scenario. For this trial, various LoRa RF configurations were tested, whilst maintaining the operation frequency at 433 MHz, as was required. Amongst these configurations, it was also possible to validate the spreading factor of 128 chips/symbol, bandwidth of 125 kHz, and coding rate 4/5 RF LoRa configuration, which was not verified in the first 433 MHz analysis. This last configuration test yielded an approximate transmission range of 5 km.

Afterwards, there was a need to verify if the LoRa transceiver communications would have any type of negative impact on the wireless communications used in the UAVs autopilot module, given the similar frequencies to the 433 MHz band. In the same location of the last trial, LoRa data packages were transmitted across the shore line whilst simultaneously transmitting with the UAVs FY-605 transceiver at 434 MHz to a computer with the GCS software. Although the FY-605 is to be used in the 462.125 MHz band, it was configured to 434 MHz to also emulate a worst-case scenario. Across the whole experiment, there was no visible interference in the GCS software or LoRa transmissions.

**Figure 13:**
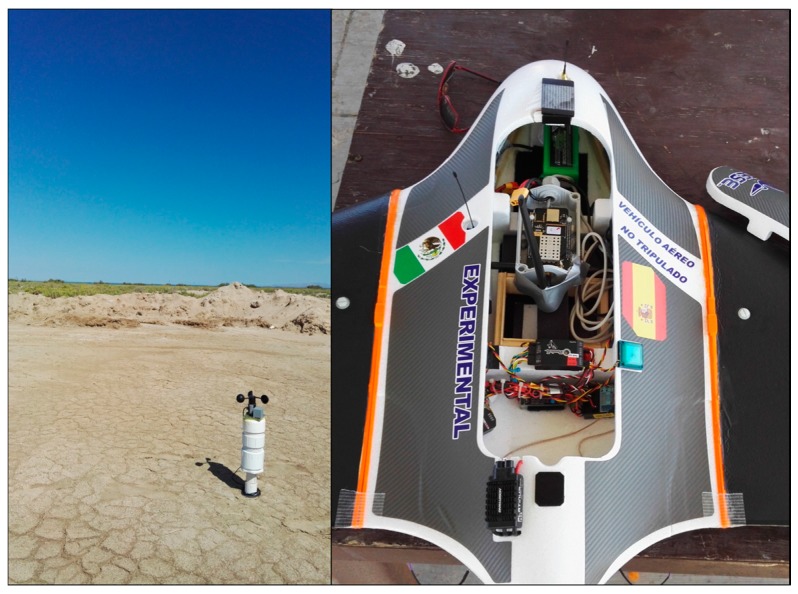
Initial flight test. To the left the sensor buoy placed on the ground and to the right the UAV with the master node mounted inside.

Even though this previous trial practically guaranteed that the LoRa master node could operate without interference, a final test was done to truly validate the communications in flight. This experiment was done using a sensor node placed on the ground, a network monitor node connected to a computer with the GUI and by mounting a master control node inside the UAV (Figure 13).

Having placed the master node in the UAV, the aircraft was flown in manual mode in close proximity to the sensor node in a circular pattern. This test granted the last successful validation of the system, prior to the final field test.

### 4.3. Field Test

**Figure 14:**
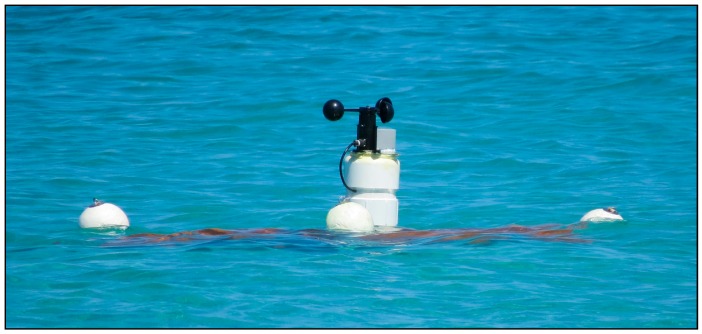
Drifting Davis buoy placed in El Tecolote (Mexico) beach for environmental data collecting.

For this last trial, a relatively controlled environment was needed to avoid any possible loss of the end devices. Thus the beach of El Tecolote (La Paz, B.C.S., Mexico) was selected, due to its ease of access and deployment. In this location, three buoys where placed approximately 60 m away from the beach (24.33657, −110.32229 UTM), with a separation of a few meters amongst each other, capturing environmental and positioning data every minute, granting high amounts of information in a short period of time (Figure 14).

Prior to their placement, all the slave nodes (sensor nodes and monitor) were configured at the 433 MHz frequency band, with a spreading factor of 128 chips/symbol, a bandwidth of 125 kHz and a coding rate 4/5, which provides a high data transfer rate at the expense of transmission range reduction, with only the minimum of noise immunity. The maximum amount of bitrate was estimated using tools and formulas provided by the transceiver´s manufacturer [44]. Although with other LoRa RF configurations increased distances are attainable, these settings allow the validation of the systems functionality as a whole.

**Figure 15:**
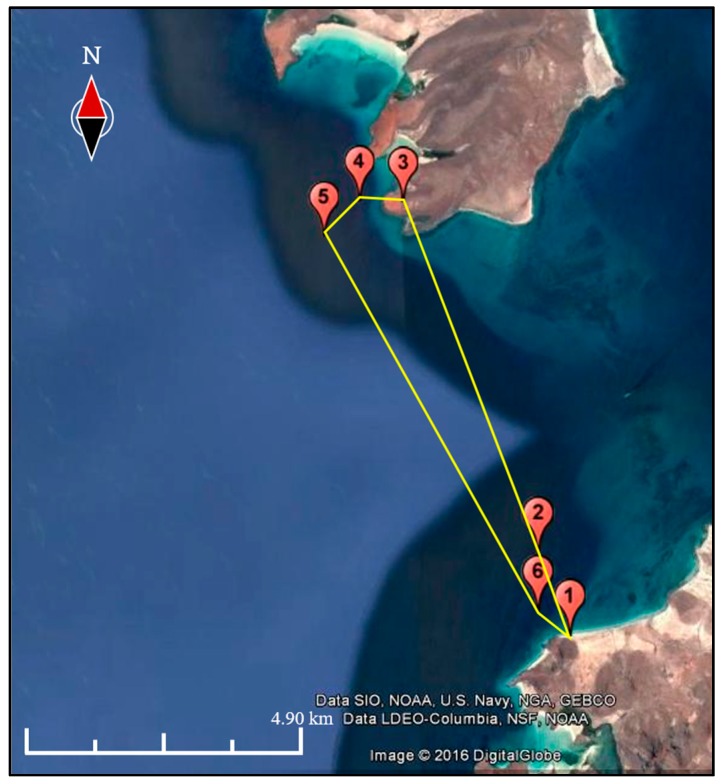
UAV flight path for the trials at El Tecolote beach. The furthest waypoint was set at 8.62 km away from the launch area and the UAV flew at a maximum height of 30 m.

To verify the proper communication between the UAV and buoys, a network monitor was placed at the beach connected to a computer. Given that the buoys and the network monitor were fairly close to each other, once the network monitor lost the reception of data from the UAV, it could be inferred that the same would happen with the buoys. After a given time in which the buoys had collected enough data, the UAV with the master node was launched from the beach, in automatic mode, following pre-established waypoints (Figure 15).

**Figure 16:**
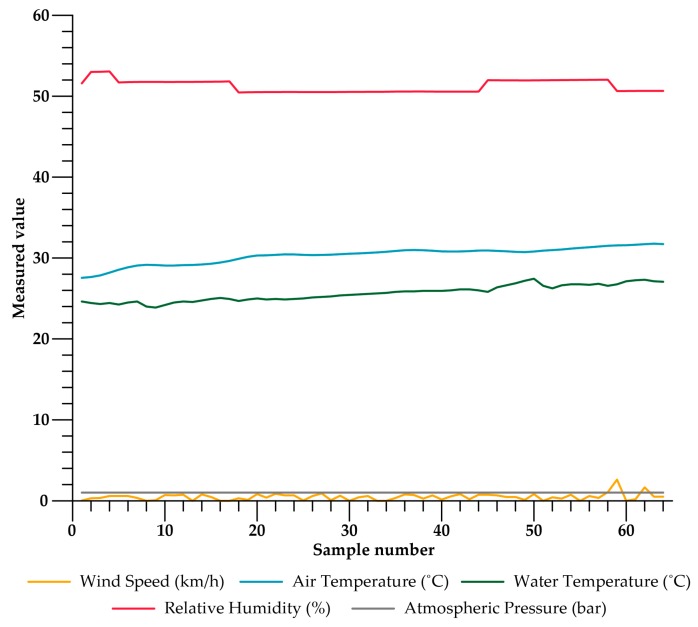
Data extracted by the master node from one of the buoys.

After the UAV had been launched, in the network monitor it could be viewed how the master node periodically located the three buoys and extracted the data. A sample of these can be seen in Figure 16.

It is worth noting that the signal from the LoRa master node was lost at approximately 4 km in distance, which is reasonable given the LoRa RF configuration set on the end devices. As verified in the pre-field tests, the maximum expected range was 5 km. Additionally, in this trial the UAV was limited to a reasonable distance, although it is able to travel double the distance with battery to spare.

The buoys, which were closely located to the beach, only drifted approximately 50 m each since the surface layer currents at that location were very weak. Yet, since there was a constant communication with the master node which was viewed with the monitor node, it can be concluded that the system as a whole is a valid one.

### 4.4. Post-Field Test Analysis

**Figure 17:**
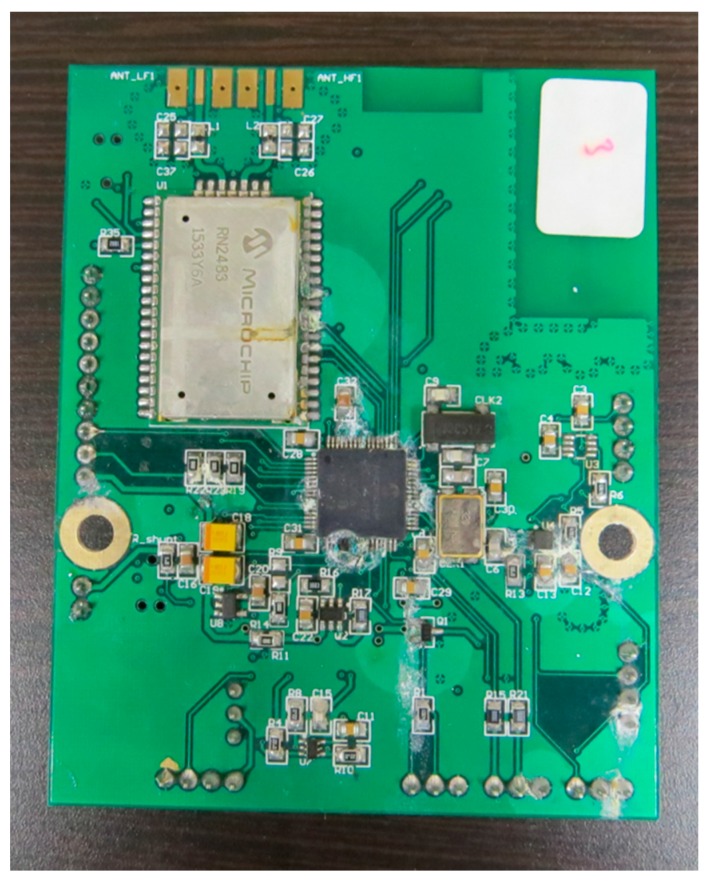
Water damaged sensor node.

After retrieving the buoys from the beach, each of these was opened to retrieve the sensor nodes placed inside. This revealed that in one of the three buoys there was a small hole in the PVC structure which allowed water to leak. Additionally, the IP67 case that housed the sensor node was missing a vital toric joint, thus water ended up filling the space where the sensor node was located (Figure 17).

This unexpected outcome suggests that the upper part of the buoy (Figure 10), although functional for the field test, requires further improvements to avoid this kind of results. Furthermore, in a small percentage of the samples extracted from the sensor nodes, a loss of GPS signal was detected. Analyzing Figure 14, it can be seen that part of the sensor node was below the sea surface, which may have been the reason for the GPS interference. Also, this might be the reason for the LoRa transmission loss at 4 km, instead of the expected 5 km. Although additional tests must be done, these results suggest that modifications should be made to the buoyancy, height of the upper part of the buoy or location of the GPS and LoRa antennas.

## 5. Discussion

Although the validation trial only tested the data extraction functional scenario, this controlled trial allowed the validation of each of the systems devices as a whole. Given that the search and rescue is the simpler functional scenario, by doing a data extraction the search and rescue was also corroborated, as the first activity in the data extraction is the localization of the slave nodes and afterwards the recollection of their data, including the GPS position.

Analyzing the results obtained in the trials, and comparing them to the proposals of Zolich et al. [27] and Barbatei et al. [28], several improvements were achieved. In terms of wireless data transmissions, the use of LoRa communications was proven to reach a notably higher transmission range of 4 km with a data rate of 5.4 kbps, which is 10 times the range achieved by Zolich et al. with an increased data rate. This also further exceeds most buoy based wireless implementations, except for satellite communications, which present additional costs. Moreover, in the work submitted by Zolich et al. there was radio interference detected with a secondary radio transmitter mounted in the quadcopter, even though this transmitter and the TinyMesh radios operated at different operating frequencies (433 MHz for the latter, 868 MHz for the former). In the validation tests carried out, it was proven that no interference was detected in the UAV’s flight communications by the LoRa transmissions, and vice versa. In terms of low power consumption, the RN2483 LoRa transceiver has a power consumption of 28.8 mA in transmission and 14.2 mA in reception, both at 3 V. In contrast with the radio used by Barbatei et al., which has a lower energy print compared to Zolich et al., the LoRa transceiver consumes less energy in both transmission and reception, even though its performance is similar. All of the previously mentioned facts translate to a lower energy consumption per data packet transmission, which in consequence increases the lifespan of a node, with a greater transmission range.

Even though the range limits of the LoRa modulation were not tested in this article, as these have already been validated in other studies [30,31,32,33,54,55], for scenarios where a greater transmission radius is desired, there are several things that must be taken into consideration for a proper deployment. Since any UAV is restricted to a finite amount of flight time, it is important to consider the bitrate of the LoRa transmissions, the frequency at which the environmental data is captured and the mode of operation of the UAV. By increasing the spreading factor of the LoRa devices the maximum transmission radius is increased, but consequently the bitrate is reduced. Furthermore, if the data capture frequency is high and the buoys have been left for an extended period of time, high amounts of data will be accumulated on each buoy. With these type of configurations it would at least be required to modify the flight plan of the UAV, first identifying the location of the buoys, then returning home and making a data extraction afterwards. An alternative to this would be to set the UAV for search and rescue mode and later extracting the data, either by collecting the buoys from their location, or to try and set a temporary base station near the buoys location with a master node (connected to a laptop) and manually download the data from the sensor nodes. It is worth noting that in a prior work [32], it was proven that by increasing the spreading factor to 256 chips/symbol transmissions could be done to a receiver station at approximately 7 km. Assuming that this same distance is achievable in a marine-coastal environment, with such a spreading factor, a bandwidth of 125 kHz and coding rate of 4/5, the bitrate would only be reduced to 3.1 kbps but effectively almost doubling the transmission radius. Such a configuration would still be viable for an elevated data capture frequency in the slave node and the mid-air data extraction with the UAV.

Another possible scenario is when the buoys are fixed to the seafloor and their location is known. Under these circumstances there are two possible ways of extracting the data from the buoys, using the UAV from a remote location to collect the data or with a master base station. The first scenario would be exactly the same as when the buoys are drifting, except that the location is already known and a more precise flight pattern can be planned. If only a master base station were to be used, the LoRa RF configurations of the nodes could be set for long-range transmissions and with high data capture frequency, with a functional behavior similar to that of current buoy implementations. The main advantage of this operation mode is the possibility of transmitting data across several kilometers, and that the base station is only formed by a master node connected to a laptop, allowing the station to be static or mobile.

Although this system completely fulfills the requirements of the SIMMA project, there are several improvements that can be made to create a more robust and functional WSN. In addition to the already mentioned enhancements made in the post-field analysis section, the inclusion of energy harvesting technologies, as proposed by Zolich et al. [27], is one of the most desirable improvements, as it would allow the buoys to monitor areas for an almost indefinite time. Also, the addition of other types of sensors and backup wireless communications, such as GSM, would grant the system with important ecological data and new ways to find marooned devices. Regarding the use of the UAV, it would be a significant improvement if the aircraft carried additional sensors, such as infrared or multispectral cameras, granting additional information that could be correlated with the data extracted from the buoys. This could be feasible with the same aircraft design, given that the UAV can carry additional payload, other than the master node, yet this was not contemplated within this article. Moreover, further integrating the UAV with the LoRa master node would be of great use, as information provided by the master node could automatically trigger different behaviors on the UAV, such as a change in flight pattern or an emergency return-to-home.

## 6. Conclusions

In this article, the design and implementation of a UAV assisted long-range buoy based WSN for the monitorization of marine-coastal environments, as well as an improvement to current implementations, has been proven. The development of a LoRa mobile wireless network, from a hardware and firmware perspective, was presented, as well as the implementation of the novel LoRaNET network protocol for LoRa devices. These two developments, applied to the SIMMA end devices, provide an easy-to-use and low power solution, with an extended range of several kilometers, as well as a more cost-effective alternative with fewer infrastructure requirements. Furthermore, it serves as a less invasive method for monitoring natural protected areas and reservoirs.

The integration of the UAV, the Davis monitoring buoys, and the LoRa communications as a single system, adds a needed flexibility to current implementations and the possibility of studying previously unreachable areas, since the buoys can be placed with a small vessel and the UAV can be flown from almost any area, given its great communications reach.

Although this proposal was aimed at marine monitoring, this same system architecture can be used for other types of environments. For instance, the upper part of the buoy, where the slave node’s electronics are housed, could be used as a portable meteorological station to temporarily monitor different areas for a given time interval.

## Figures and Tables

**Table 1:** Network device commands implemented in LoRaNET.

Category	Name	Description
Commands for slave nodes which are not in a network	Recruiting	Message used by the master node to convey to the other nodes that they are allowed to join the newly created network
	Request to join	Nodes that received the recruiting message may request to join the network
	Response to a join	Answer given to a slave node from a master node after a request to join
	Beacon	Slave nodes that are not in a network may send this message to transmit their location or data relevant to the application
Commands associated to network maintenance tasks	Ping	Transmits a ping to a slave node in the network
	Request to leave net	The master node requests a slave node to leave the network
	Event leave network	Notification message of a node that left the network
	Scan network	The master node sends a scan network petition to the 0xFFFF address (broadcast), to which any node in the network answers
	Synchronism request	The master node transmits its time (GPS) to all nodes in the network
Commands associated to messaging	MAC ACK	Message sent as a confirmation of message reception
	Data message	Main data message, with a maximum payload of 128 bytes
